# The putative small terminase from the thermophilic dsDNA bacteriophage G20C is a nine-subunit oligomer

**DOI:** 10.1107/S1744309113017016

**Published:** 2013-07-27

**Authors:** Juan Loredo-Varela, Maria Chechik, Vladimir M. Levdikov, Ahmad Abd-El-Aziz, Leonid Minakhin, Konstantin Severinov, Callum Smits, Alfred A. Antson

**Affiliations:** a York Structural Biology Laboratory, Department of Chemistry, University of York, York YO10 5DD, England; b Waksman Institute for Microbiology, Rutgers, The State University of New Jersey, Piscataway, NJ 08854, USA; c Department of Molecular Biology and Biochemistry, Rutgers, The State University of New Jersey, Piscataway, NJ 08854, USA; d Institutes of Molecular Genetics and Gene Biology, Russian Academy of Sciences, Moscow 119334, Russian Federation

**Keywords:** putative small terminase, *Thermus thermophilus*, bacteriophage G20C

## Abstract

The assembly of double-stranded DNA bacteriophages is dependent on a small terminase protein that normally plays two important roles. Firstly, the small terminase protein specifically recognizes viral DNA and recruits the large terminase protein, which makes the initial cut in the dsDNA. Secondly, once the complex of the small terminase, the large terminase and the DNA has docked to the portal protein, and DNA translocation into a preformed empty procapsid has begun, the small terminase modulates the ATPase activity of the large terminase. Here, the putative small terminase protein from the thermostable bacteriophage G20C, which infects the Gram-negative eubacterium *Thermus thermophilus*, has been produced, purified and crystallized. Size-exclusion chromatography–multi-angle laser light scattering data indicate that the protein forms oligomers containing nine subunits. Crystals diffracting to 2.8 Å resolution have been obtained. These belonged to space group *P*2_1_2_1_2_1_, with unit-cell parameters *a* = 94.31, *b* = 125.6, *c* = 162.8 Å. The self-rotation function and Matthews coefficient calculations are consistent with the presence of a nine-subunit oligomer in the asymmetric unit.

## Abstract 2

The putative small terminase protein from the thermostable bacteriophage G20C has been produced, purified and crystallized.

## 1. Introduction

During the assembly of double-stranded DNA bacteriophages, DNA is usually translocated into preformed procapsids by a molecular motor consisting of the small and large terminase proteins and the portal protein (Casjens, 2011 ▶; Feiss & Rao, 2012 ▶). The portal protein, a circular oligomer embedded in one of the fivefold symmetrical vertices of the icosahedral shell, contains a tunnel for DNA translocation. Initially, the small terminase specifically recognizes the bacteriophage genomic DNA and recruits the large terminase protein. Following DNA cutting by the large terminase, the complex of the two terminase proteins and DNA docks to the portal protein of an empty prophage. DNA translocation into the procapsid is fuelled by the ATPase activity of the large terminase protein (Sun *et al.*, 2008 ▶). During DNA translocation, the small terminase protein modulates the ATPase and nuclease activities of the large terminase protein (Gual *et al.*, 2000 ▶).

Three-dimensional structural data are available for oligomeric assemblies of small terminases from several viruses including Sf6, SF6, T4 and P22 (Zhao *et al.*, 2010 ▶; Büttner *et al.*, 2012 ▶; Sun *et al.*, 2012 ▶; Roy *et al.*, 2012 ▶). All were shown to assemble into ring-like structures composed of 8–12 subunits arranged symmetrically around a central axis. The main topological domains identified in the small terminase are (i) the C-terminal oligomerization domain, which establishes inter-subunit contacts around a central tunnel, and (ii) the exposed N-terminal domain, which in phages SPP1 and SF6 binds to the recognition *pac* site DNA (Büttner *et al.*, 2012 ▶). Interestingly, in phage P22 the DNA-binding function is attributed to a short segment at the C-terminus (Roy *et al.*, 2012 ▶).

Despite the availability of structural information, several questions concerning the mechanism by which the small terminase carries out its function remain to be answered. As mentioned above, there appear to be differences in the way that different terminases recognize the genomic DNA (Büttner *et al.*, 2012 ▶; de Beer *et al.*, 2002 ▶; Sun *et al.*, 2012 ▶; Roy *et al.*, 2012 ▶). Perhaps more importantly, there are contradicting reports about the potential involvement of the central tunnel in DNA translocation (Roy *et al.*, 2012 ▶; Büttner *et al.*, 2012 ▶).

To answer some of these questions, we initiated structural and functional studies on the putative small terminase protein from the *Thermus thermophilus* bacteriophage G20C, which is a close relative of bacteriophages P23-45 and P74-26 (Minakhin *et al.*, 2008 ▶). Here, we report the production of recombinant protein in *Escherichia coli*, protein purification and crystallization. The results of size-exclusion chromatography coupled with multi-angle laser light scattering (SEC–MALLS) and crystal data indicate that the protein forms nine-subunit oligomers, like the small terminases found in bacteriophages SF6 and P22 (Büttner *et al.*, 2012 ▶; Roy *et al.*, 2012 ▶).

## 2. Materials and methods

### 2.1. Cloning

It was not possible to locate the small terminase gene based on sequence homology to small terminases from other viruses. However, as the small terminase is usually encoded by a gene immediately preceding the large terminase and portal protein genes, and because the corresponding G20C gene had an appropriate size, we decided to clone this gene of G20C. This gene corresponds to the ORF P23p84 (UniProtKB/TrEMBL A7XXB6) in the closely related phage P23-45 (Minakhin *et al.*, 2008 ▶). Forward and reverse primers containing the *Nde*I and *Hin*dIII restriction-site sequences, respectively, enclosing the full-length protein were designed as follows: forward, 5′-GGA­CAACATATGAGCGTGAGTTTTAGGGAC-3′; reverse, 5′-GGCAAGCTTCTAGGTCTTAGGCGCTTCATC-3′. The amplified segment was cloned into the pET28a vector (Novagen, Merck KGaA).

### 2.2. Protein expression and purification

All chemicals were purchased from Sigma–Aldrich, unless stated otherwise. *E. coli* B834 cells (Novagen, Merck KGaA) were transformed with the recombinant DNA and grown at 310 K until the OD_600_ reached ∼0.8. Protein expression was then induced with 1 m*M* IPTG at 289 K. Before sonication, the cells were lysed in a buffer consisting of 500 m*M* NaCl, 50 m*M* Tris pH 7.5, 20 m*M* imidazole, 100 µg ml^−1^ lysozyme, 0.7 µg ml^−1^ pepstatin A, 0.5 µg ml^−1^ leupeptin, 100 m*M* 4-(2-aminoethyl)benzenesulfonyl fluoride hydrochloride. The His-tagged protein was purified by Ni-affinity chromatography (ÄKTA, GE Healthcare) by binding the protein to nickel beads on a His-Trap column (GE Healthcare) and by further elution with an imidazole gradient. The binding and elution buffers consisted of 500 m*M* NaCl, 50 m*M* Tris pH 7.5 with 20 and 500 m*M* imidazole, respectively.

The His tag was cleaved by thrombin digestion (BD Biosciences) while the sample was dialysed against the binding buffer (no imidazole). One unit of thrombin per milligram of protein was used to digest the protein overnight. A second Ni-affinity chromatography was performed to separate cleaved protein from noncleaved protein, followed by size-exclusion chromatography in 250 m*M* NaCl, 20 m*M* Tris pH 7.5 using a Superdex 16/60 column (GE Healthcare).

### 2.3. Characterization of the oligomeric state by SEC–MALLS

Thrombin-digested protein was diluted to 4 mg ml^−1^ in 250 m*M* NaCl, 20 m*M* Tris pH 7.5 and loaded onto a BioSep SEC-s3000 gel-filtration column (Phenomenex) which was equilibrated with 250 m*M* NaCl, 20 m*M* Tris pH 7.5. Size-exclusion chromatography was carried out on a Shimadzu HPLC system with a flow rate of 0.5 ml min^−1^. The elution was monitored at 280 nm using a SPD20A UV–Vis detector. Light-scattering data were recorded by a Dawn HELEOS II 18-angle light-scattering detector and the concentration of the eluting protein was measured using an inline Optilab rEX refractive-index monitor (Wyatt Technology). Data were analysed using the *ASTRA V* software package (Wyatt Technology).

### 2.4. Crystallization

The purified protein was concentrated to 21 mg ml^−1^ in a solution containing 175 m*M* NaCl and 10 m*M* Tris pH 7.5. Crystallization experiments using the Index screen (Hampton Research) were set up with a Mosquito nanolitre pipetting robot (TTP LabTech). Crystals grew within a few days from condition No. 80 of the Index screen in sitting drops at 293 K. These conditions were manually optimized in 24-well hanging-drop plates (Greiner Bio-One) and the obtained crystals were used as seeds for subsequent optimization experiments. A seed stock was produced using a tube with a seed bead (Hampton Research) and was stored in 100 µl mother liquor consisting of 0.4 *M* ammonium acetate, 0.1 *M* HEPES pH 7.5, 30%(*w*/*v*) PEG 3350. 0.5 µl of the seed stock was mixed with 1 µl protein solution and 1 µl mother liquor for the next round of optimization. The best crystals grew within one month using a reservoir solution consisting of 0.4 *M* ammonium acetate, 23%(*w*/*v*) PEG 3350, 0.1 *M* HEPES pH 7.5, 9%(*v*/*v*) ethylene glycol. Crystals were tested in-house using an MSC MicroMax-007 HF rotating-anode X-ray generator (Rigaku) and a MAR345 detector (MAR Research).

### 2.5. X-ray data collection and processing

X-ray data were collected from a single cryocooled crystal on the I04 beamline at the Diamond Light Source, England. Data were collected at a wavelength of 0.9200 Å with a crystal-to-detector distance of 325.2 mm, a 0.2° crystal rotation per image and a total crystal rotation range of 180°. The data were indexed with *HKL*-2000 (Otwinowski & Minor, 1997 ▶) and processed with *XDS* (Kabsch, 2010 ▶) and *SHELX* beta (Sheldrick, 2010 ▶). The self-rotation function was calculated using *MOLREP* (Vagin & Teplyakov, 2010 ▶) with a resolution range of 10–3.0 Å and a radius of integration of 35 Å. Other crystallographic calculations were performed using the *CCP*4 suite of programs (Winn *et al.*, 2011 ▶).

### 2.6. Secondary-structure prediction

The secondary structure of the putative small terminase was predicted using *Jpred* (Cole *et al.*, 2008 ▶).

## 3. Results and discussion

### 3.1. Cloning, protein expression and purification

**Figure 1:**
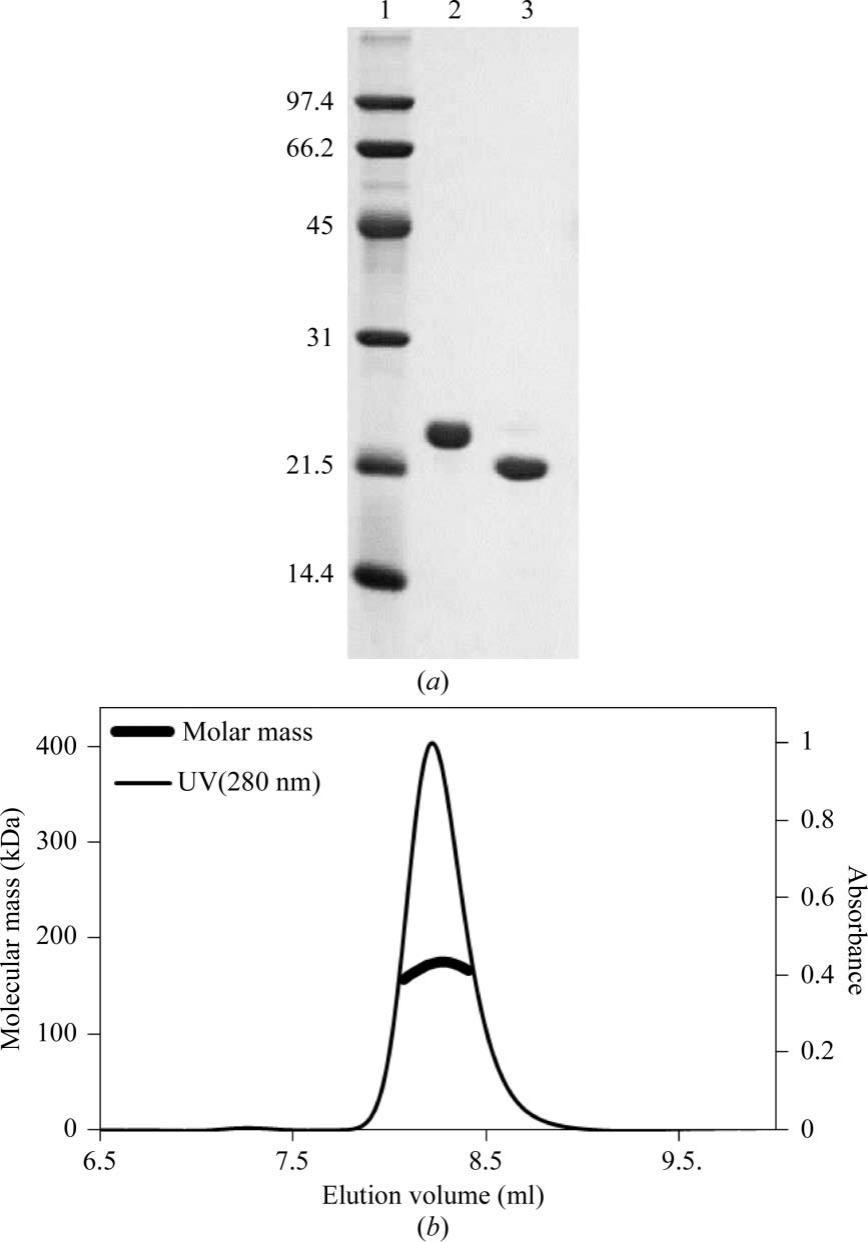
Protein purification and characterization. (*a*) SDS–PAGE showing undigested (lane 2) and thrombin-digested (lane 3) samples. Lane 1 contains molecular-mass markers (labelled in kDa). (*b*) SEC–MALLS analysis. The thin line corresponds to the absorbance at 280 nm. The thick line below the absorbance peak corresponds to the molecular weight calculated for the eluted protein.

The recombinant protein, containing a His tag at the N-terminus with a thrombin protease cleavage site between the tag and the protein-coding region, was expressed in *E. coli* B834 cells at 289 K. The molecular weight of the expressed protein was 20 957 Da, or 19 074 Da after thrombin cleavage. Following thrombin digestion, size-exclusion chromatography produced a highly purified protein sample (Fig. 1 ▶ *a*).

### 3.2. Oligomeric state determination by SEC–MALLS

**Figure 2:**
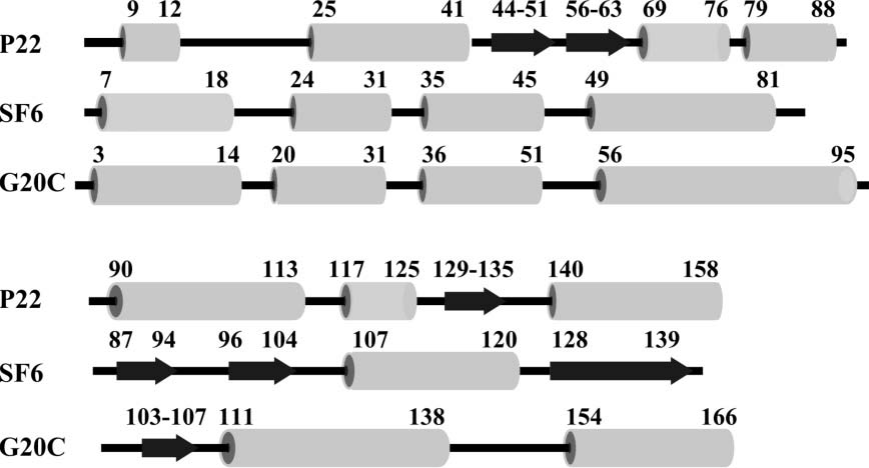
Secondary-structure alignment for small terminase proteins from bacteriophages SF6 and P22 and the putative G20C small terminase.

SEC–MALLS analysis was performed to assess whether the protein forms oligomers containing multiple subunits, as observed for the small terminases of other phages. This experiment was performed with the final purified protein sample following thrombin digestion and the second Ni-affinity purification. The data revealed a homogeneous monodisperse protein preparation with an estimated molecular mass of ∼170.9 kDa, corresponding to 8.9 subunits per oligomer (Fig. 1 ▶ *b*). The data indicate that the putative small terminase protein forms nine-subunit oligomers, as observed for the small terminases of bacteriophages SF6 (Büttner *et al.*, 2012 ▶) and P22 (Roy *et al.*, 2012 ▶). The predicted secondary structure of the G20C protein is consistent with the secondary structure of these two small terminases (Fig. 2 ▶). Interestingly, while the four N-terminal α-helices match the secondary structure observed in the SF6 small terminase, the α-­helices at the C-terminus are more consistent with the secondary structure observed in the P22 protein.

### 3.3. Crystallization and X-ray data analysis

**Figure 3:**
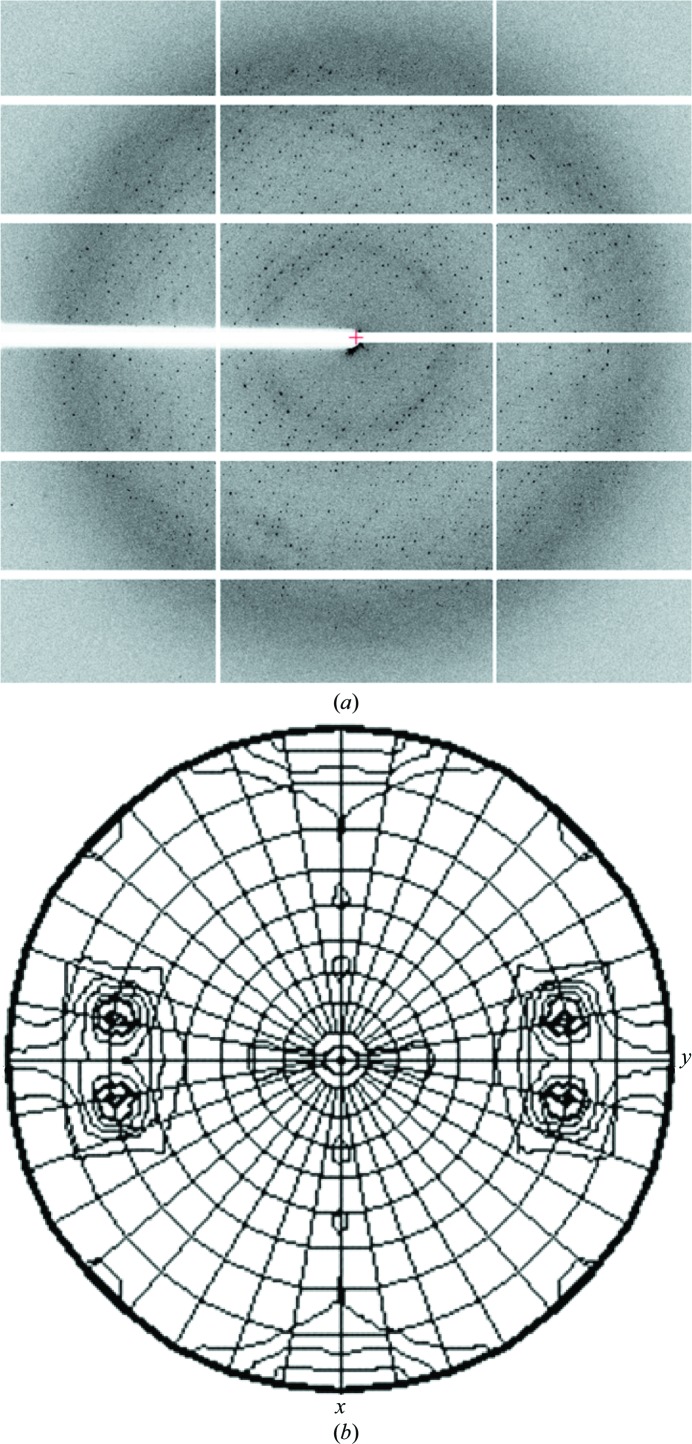
X-ray analysis. (*a*) Diffraction image. The resolution at the edge of the plate is 2.5 Å. (*b*) Stereographic projection (κ = 40° section) of the self-rotation function.

The best crystals were obtained by microseeding using 21 mg ml^−1^ protein solution containing 175 m*M* NaCl and 10 m*M* Tris pH 7.5 and reservoir solution consisting of 0.4 *M* ammonium acetate, 23%(*w*/*v*) PEG 3350, 0.1 *M* HEPES pH 7.5, 9%(*v*/*v*) ethylene glycol. The synchrotron X-ray data from a crystal belonging to the orthorhombic space group *P*2_1_2_1_2_1_, with unit-cell parameters *a* = 94.31, *b* = 125.6, *c* = 162.8 Å, extended to 2.8 Å resolution (Fig. 3 ▶ *a*, Table 1 ▶).

The highest peaks in the self-rotation function calculated with *MOLREP* were 17% of the origin peak. These peaks are in the κ = 40° section, corresponding to ninefold rotational symmetry (Fig. 3 ▶ *b*). The specific volume, corresponding to nine subunits per asymmetric unit, is 2.8 Å^3^ Da^−1^. This corresponds to a solvent content of 56.1% (Winn *et al.*, 2011 ▶; Matthews, 1968 ▶).

Although both SEC–MALLS and crystallographic data indicate nine-subunit oligomers, as observed for the P22 and SF6 small terminases (Roy *et al.*, 2012 ▶; Büttner *et al.*, 2012 ▶), structure determination by molecular replacement is not possible owing to a complete lack of sequence similarity. The next stage of this project will focus on experimental phasing.

## 4. Conclusions

The putative small terminase protein from the thermophilic bacteriophage G20C forms nine-subunit assemblies both in solution and in the crystal. The genomic context, the predicted secondary structure and the oligomeric state of the protein are consistent with this protein being the small terminase.

## Figures and Tables

**Table 1:** X-ray data statistics Values in parentheses are for the outermost resolution shell.

X-ray source	I04, Diamond Light Source
Wavelength (Å)	0.92000
Temperature (K)	100
Space group	*P*2_1_2_1_2_1_
Unit-cell parameters (Å)	*a* = 94.3, *b* = 125.6, *c* = 162.8
Resolution range (Å)	49.8–2.8 (2.89–2.80)
No. of unique reflections	48371 (4371)
*R*_merge_ † (%)	9.6 (15.3)
Average *I*/σ(*I*)	16.3 (1.3)
Completeness (%)	99.9 (100)
Multiplicity	6.8 (6.1)

† *R*_merge_ = 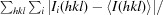 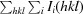, where *I*_*i*_(*hkl*) is the intensity of the *i*th observation of reflection *hkl*, 〈*I*(*hkl*)〉 is the average value of the intensity, the sum 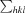 is over all measured reflections and the sum 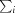 is over *i* measurements of a reflection.
